# The risk of malaria in Ghanaian infants born to women managed in pregnancy with intermittent screening and treatment for malaria or intermittent preventive treatment with sulfadoxine/pyrimethamine

**DOI:** 10.1186/s12936-016-1094-z

**Published:** 2016-01-28

**Authors:** Timothy Awine, Mark M. Belko, Abraham R. Oduro, Sunny Oyakhirome, Harry Tagbor, Daniel Chandramohan, Paul Milligan, Matthew Cairns, Brian Greenwood, John E. Williams

**Affiliations:** Navrongo Health Research Centre, PO Box 114, Navrongo, Ghana; Novartis Pharma Services, Lagos, Nigeria; Department of Community Medicine, Kwame Nkrumah University of Science and Technology, Kumasi, Ghana; Faculty of Infectious and Tropical Diseases, London School of Hygiene and Tropical Medicine, London, UK; Faculty of Epidemiology and Population Health, London School of Hygiene and Tropical Medicine, London, UK

**Keywords:** Malaria in infants, Placental malaria, Intermittent screening and treatment in pregnancy, Intermittent preventive treatment in pregnancy

## Abstract

**Background:**

Several studies have reported an association between malaria infection of the placenta and the risk of malaria in young children in the first year of life, but it is not known if this is causal, or influenced by malaria control measures during pregnancy. This paper compares the incidence of malaria in infants born to mothers who received either intermittent preventive treatment with sulfadoxine/pyrimethamine (IPTp-SP) or screening with a rapid diagnostic test and treatment with artemether–lumefantrine (ISTp-AL) during their pregnancy.

**Methods:**

From July 2011 to April 2013, 988 infants of women enrolled in a trial of IPTp-SP versus ISTp-AL in the Kassena-Nankana districts of northern Ghana were followed to determine the risk of clinical malaria during early life, and their risk of parasitaemia and anaemia at 6 and 12 months of age. In addition, the incidence of clinical malaria in infants whose mothers had malaria infection of the placenta was compared with that in infants born to women free of placental malaria.

**Results:**

The incidence of clinical malaria was 0.237 and 0.211 episodes per child year in infants whose mothers had received ISTp-AL or IPTp-SP, respectively. The adjusted incidence rate ratio and the adjusted rate difference were 0.94 (95 % CI 0.68, 1.33) and 0.029 (95 % CI −0.053, 0.110) cases per child year at risk respectively. The incidence of clinical malaria was similar in infants born to women with placental malaria (0.195 episodes per child year) and in infants of women without placental malaria (0.224 episodes per child year) (rate ratio = 0.86 [95 % CI 0.54, 1.37]).

**Conclusion:**

Infants born to women managed with ISTp-AL during pregnancy were not at greatly increased risk of malaria compared with infants born to women who had received IPTp-SP. The incidence of malaria in infants was similar whether or not their mother had had placental malaria.

**Electronic supplementary material:**

The online version of this article (doi:10.1186/s12936-016-1094-z) contains supplementary material, which is available to authorized users.

## Background

Malaria infection during pregnancy is a major public health concern because of the risks malaria poses not only to the mother but also to her infant [1]. Some studies have shown that malaria infection during pregnancy, particularly in the last trimester, is associated with an increased risk of malaria in infants. A clinical episode of malaria during pregnancy has been associated with a doubling of risk and malaria infection of the placenta (PM) with a four-fold increase in risk [2–4]. However, these associations have not been shown to be causal [5] and may reflect confounding by different levels of exposure [6], confounding which is likely to be very strong given the well-documented heterogeneity in malaria risk. PM infection may also increase an infant’s susceptibility to malaria-related co-morbidities, including all-cause anaemia during early childhood, which has been reported to be three times more prevalent among infants born to women with placental parasitaemia than among infants born to non-infected women [7–12]. Anti-malarial interventions that influence the likelihood of malaria infection during pregnancy may, therefore, have an effect on infants during early life. Consequently, evaluation of the impact of novel anti-malarial interventions during pregnancy should include study of their impact not only on pregnant women but also on their infants.

Intermittent preventive treatment with sulfadoxine/pyrimethamine (IPTp-SP) is one of the WHO’s recommended methods for prevention of malaria during pregnancy [13]. However, this intervention may be threatened by increasing resistance to SP in eastern and southern Africa [14] and IPTp-SP may not be an appropriate intervention in areas of low transmission where most women who receive IPTp-SP will not be at risk. Screening of women at routine antenatal clinics with a rapid diagnostic test (RDT) and treatment of those who are positive with an artemisinin-based combination therapy (ACT), an approach termed intermittent screening and treatment (ISTp), is an alternative approach to IPTp-SP that is being evaluated. A pilot trial of ISTp using artemether–lumefantrine (ISTp-AL) in Ghana [15], and a further trial in four West African countries (Burkina Faso, Ghana, Mali, The Gambia) where *Plasmodium falciparum* is still sensitive to SP [16], have shown that ISTp-AL is non-inferior to IPTp-SP in preventing low birth weight, maternal anaemia and PM. However, it is not known if infants whose mothers were managed with ISTp-AL during pregnancy might be at increased risk of malaria and co-morbidities during their first year of life compared to infants born to mothers who received IPTp-SP. Therefore, this study investigated the incidence of malaria, anaemia and co-morbidities in infants born to women in Kassena-Nankana district, northern Ghana, who participated in a multicentre trial of ISTp-AL versus IPTp-SP.

## Methods

### Study site

The study was conducted in the Kassena-Nankana East municipal and Kassena-Nankana West Districts of the Upper East Region of Ghana, where malaria transmission is intense and highly seasonal. Rainfall occurs mainly between May and October and averages between 800 and 1000 mm per year. The mean monthly temperature range is 20–45 °C. Transmission by *Anopheles gambiae* and *Anopheles funestus* peaks at the end of the wet season (October and November) [17–20]. The entomological inoculation rate (EIR) is higher in irrigated areas than in non-irrigated zones with values reaching as high as 400 infective bites/person-year in 2001-02 [4]. *Plasmodiumfalciparum* is the dominant malaria parasite and is still sensitive to SP [16]. HIV prevalence in the study area is low (<2 %).

### Study population

The study population comprised infants born to women managed with IPTp-SP or ISTp-AL during their pregnancy in the course of an individually randomized, controlled trial of these two interventions. Infants who lived in the study area, whose mothers had received IPTp-SP or ISTp-AL at least once (i.e., received SP in the IPTp-SP group, or were screened at least once for malaria with an RDT and treated with AL if found to be positive in the ISTp-AL group) and whose mothers or guardians gave consent were eligible for enrolment in the follow-up study. For logistic reasons the study start was delayed and follow-up was not initiated immediately after a woman had delivered in all children. Therefore, the age at enrolment ranged from nought to 6 months.

### Recruitment and follow-up

The duration of follow-up was 12 months so that, depending on age at recruitment, infants were followed up until between 12 and 18 months of age. During the follow-up period, information on incidence of both symptomatic malaria and other illnesses was obtained during unscheduled visits when children reported ill to one of the health facilities within the study area. Children who presented with symptoms or signs suggestive of malaria were screened using a RDT to guide treatment, subsequently the diagnosis was confirmed by microscopy, and haemoglobin (Hb) was measured. Blood samples for testing malaria parasitaemia and Hb were also obtained at prescheduled visits at the health centres, which occurred at 6 and 12 months of age.

### Laboratory procedures

Thick and thin film blood smears were prepared as described previously [21]. Slides were read twice by two independent readers to identify malaria parasites and to quantify their density. Slides were considered negative if no parasites were seen in 100 high power fields of a thick blood film. If a slide was judged to be discordant, (one positive and the other negative, or a two-fold or more difference in parasite density was found between readers), a third independent reading was made to resolve the discordant results. In the case of discordance on positivity/negativity, the majority view was taken. Haemoglobin concentration (g/dL) was measured using a HemoCue^®^ Hb 301 system (HemoCue AB, Sweden). Moderate anaemia was defined with a cut-off at 11 g/dL. PM was determined by histological examination of the placenta, as described in Tagbor et al. [16]. PM infection was defined as active infection, i.e., parasites seen with or without malaria pigment present.

### Data management and statistical analyses

**Fig. 1:**
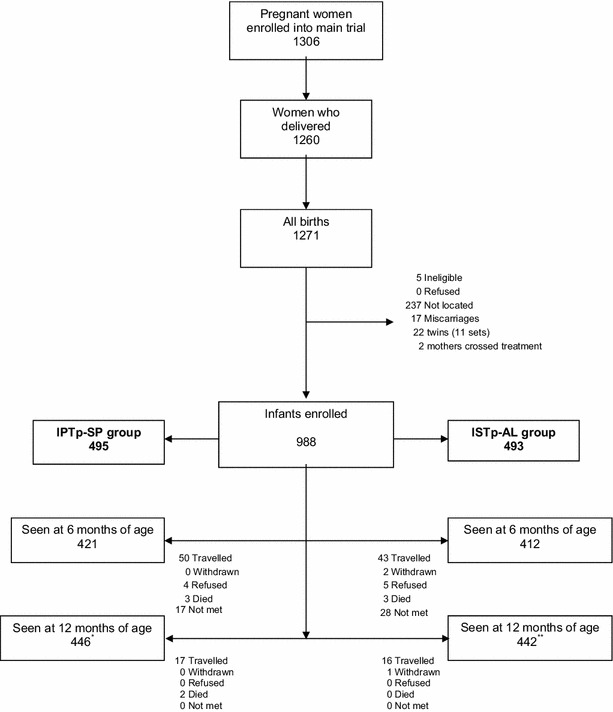
Trial profile: number of infants enrolled and numbers seen at age 6 and 12 months of age surveys-ATP1 population. *44 of these infants seen at 12 months of age were not seen at 6 months of age in the IPTp-SP group. **46 of these infants seen at 12 months of age were not seen at 6 months of age in the ISTp-AL group

Clinical data were captured on structured case report forms (CRFs) and double entered into a database designed using Epidata 3.0 software, and verified. All statistical analyses were carried out using Stata version 13.1 (StataCorp, College Station, TX, USA). The primary outcome for the study was the incidence of clinical malaria, defined as a temperature ≥37.5 °C or a history of fever within the past 48 hours and the presence of *P.falciparum* parasitaemia of any density. Sample size for the study was constrained by the number of women enrolled in Ghana in the multicentre trial. A total of 1306 women were enrolled in the main study, 1260 were followed to delivery and 988 infants were enrolled and included in the analysis (Fig. 1).

The primary analysis (According to Protocol, ATP1) included infants seen on at least one of the two scheduled visits (at 6 or 12 months of age) and whose mothers received at least two courses of SP (if they were in the IPTp-SP group) or were screened with an RDT at least twice (if in the ISTp-AL group). A secondary analysis (ATP2) included all children who were followed during the study period, regardless of the number of courses of intervention their mothers had received (although mothers of all children recruited in the infant study had received at least one course of the appropriate intervention), and regardless of the number of visits recorded. Twins and two children whose mothers had received a mixture of interventions (one from the IPTp-SP group who had been screened and treated at an antenatal clinic (ANC), and one from the ISTp-AL group, who had received SP in error) were excluded from both analyses.

For the primary outcome, rate ratios and 95 % confidence intervals were obtained using a Poisson regression model with gamma-distributed random effect to account for the within-child correlation between malaria episodes. Adjusted rate differences were estimated using the ordinary least squares regression approach developed by Xu et al. [22]. Risk ratios and risk differences were calculated for clinical malaria using the approaches proposed by Zou [23] and Cheung et al. [24], respectively. Other secondary outcomes were analysed using similar methods. Covariates that were found to have a confounding effect were included in the multivariable models to obtain adjusted estimates. Additional covariates that were thought to have an important association with the incidence of malaria *a priori* were also included. These covariates were season (November to May as dry and June to October as wet), residence in an irrigated or non-irrigated area, rural or urban residence location, treated bed net use (use of bed net always), and socio-economic status (based on household assets using quintiles generated by Principal Components Analysis (PCA) of the mother. In the analysis comparing IPTp-SP and ISTp-AL, mother’s PM status was not included as a covariate, because this variable was missing for 302 infants and because, if there is a causal relationship between intervention group and incidence in infancy, PM is likely to be on the causal pathway.

### Ethics

The study was approved by the Navrongo Health Research Centre Institutional Review Board, the Committee on Human Research, Publication and Ethics of the Kwame Nkrumah University of Science and Technology and Komfo Anokye Teaching Hospital, both in Kumasi, Ghana as well as by the London School of Hygiene and Tropical Medicine Ethics Committee. Written informed consent was obtained from all women on behalf of their offspring. The conduct of the trial was guided by the Data Safety and Monitoring Board. The clinical trial of ISTp-AL *versus* IPTp-SP in pregnant women was registered on clinical trials.gov [NCT01084213].

## Results

A total of 988 infants were enrolled (493 in the ISTp-AL group and 495 in the IPTp-SP group (Fig. 1). The ATP1 population was 440 in the ISTp-AL group and 442 in the IPTp-SP group. The baseline characteristics of infants in the two groups were similar with respect to gender, season of birth, use of an insecticide-treated bed net (ITN), place of residence (urban/rural) and socio-economic status of the household (Table 1). The characteristics of their mothers (gravidity, number of doses of ISTp-AL or IPTp-SP received and the age at delivery) were also similar in the two study groups. Additional file 1 shows the characteristics of children and mothers enrolled and not enrolled, which were also similar. Number of children and duration of follow-up by age at enrolment is also shown on Additional file 2.

**Table 1:** Baseline characteristics of study mothers and children

Characteristics	IPTp-SP n (495)	ISTp-AL n (493)
*Maternal*		
Gravidity, n (%)		
Primigravidae	269 (54.3)	256 (52.1)
Secundigravidae	226 (45.7)	235 (47.9)
Number of SP courses (IPTp-SP) or number of times screened (ISTp-AL arm), n (%)		
1	40 (8.1)	30 (6.1)
2	130 (26.3)	144 (29.2)
3	325 (65.7)	319 (64.7)
Placental malaria (PM), n^a^ (%)		
PM +ve	106 (30.5)	96 (28.3)
PM –ve	241 (69.5)	243 (71.7)
Marital status, n (%)		
Married	430 (86.9)	436 (88.4)
Single	64 (12.9)	55 (11.2)
Other	1 (0.2)	2 (0.4)
Age at delivery [mean (SD)], years	22.3 (3.7)	22.4 (3.8)
SES, n^a^ (%)		
Least poor	55 (11.2)	57 (11.6)
Less poor	69 (14.0)	53 (10.8)
Poor	114 (23.1)	96 (19.5)
More poor	169 (34.3)	184 (37.4)
Most poor	86 (17.4)	102 (20.7)
*Child*		
Gender, n (%)		
Male	230 (46.5)	235 (47.7)
Female	265 (53.5)	258 (52.3)
Birth season, n (%)		
Wet (June to October)	225 (45.5)	220 (44.6)
Dry (November to May)	270 (54.6)	273 (55.4)
ITN use^b^, n^a^ (%)		
Yes	370 (74.9)	352 (71.4)
No	124 (25.1)	141 (28.6)
Residence location, n (%)		
Urban	61 (12.3)	47 (9.5)
Rural	434 (87.7)	446 (90.5)
Live in irrigation area, n (%)		
Yes	52 (10.5)	66 (13.4)
No	443 (89.5)	427 (86.6)
Age at enrolment, months (SD)	4.9 (2.6)	4.8 (2.6)
Birth weight [mean(SD)], kg	2.8 (0.4)	2.8 (0.4)

*PM* placental malaria, *IPTp-SP* intermittent preventive treatment with sulfadoxine/pyrimethamine, *ISTp-AL* screening with a rapid diagnostic test (RDT) and treatment with artemether–lumefantrine, *SES* socio-economic status, *ITN* insecticide-treated bed net, *SD* standard deviation

^a^Missing: PM: IPTp-SP = 148, ISTp-AL = 154; SES:IPTp-SP = 2, ISTp-AL = 1; ITN use:IPTp-SP *=* 1, ISTp-AL = 0

^b^Child reported to regularly sleep under an ITN since last visit

**Fig. 2:**
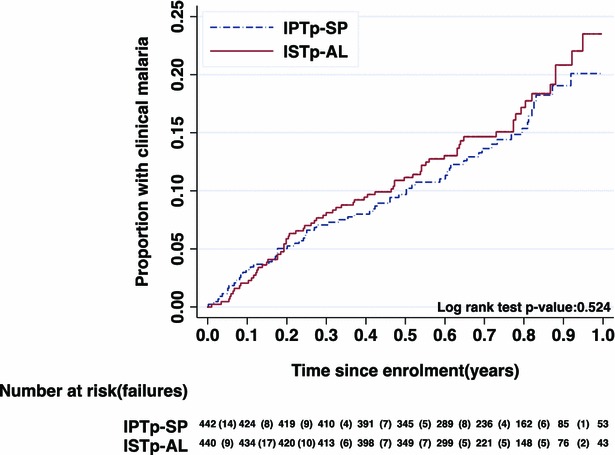
Cumulative incidence of clinical malaria episode by follow-up time—ATP1 population

In the ATP1 population, 73 and 66 episodes of clinical malaria were detected in the ISTp-AL and IPTp-SP groups, respectively; the corresponding figures for the ATP2 population were 77 and 72 episodes, respectively (Table 2). Children of women in the ISTp-AL group experienced a slightly higher incidence rate of clinical malaria compared to those of women in the IPTp-SP group, (0.24 episodes per year [95 % CI 0.19, 0.30] and 0.21 [95 % CI 0.17, 0.27]), respectively (Fig. 2). The incidence rate ratio and rate difference were 1.11 [95 % CI 0.78, 1.59] and 0.026 [95 % CI −0.053, 0.104], respectively. After adjusting for infant’s gender, socio-economic status of the household, rural/urban residence, residence in an irrigated area, season, ITN use, infant’s age at visit, mother’s parasitaemia status on the day she was enrolled into the main trial and pre-delivery Hb concentration, the incidence rate ratio was 0.94 [95 % CI 0.68, 1.33] (Table 2). The adjusted rate difference was slightly higher incidence in the ISTp group, 0.029 [95 % CI −0.053, 0.110] (Table 2).

**Table 2:** Incidence of episodes of clinical malaria in study children (all episodes during passive surveillance)

Analysis population, Intervention group	Clinical malaria episodes	Person-years at risk	Incidence rates per year	Rate ratio^a^ (95 % CI)	p value*
ATP1, IPTp-SP	66	312.7	0.21	(Reference)	–
ATP1, ISTp-AL	73	308.5	0.24	0.94 (0.68, 1.33)	0.76
				Rate difference^a^ (95 % CI)	p value
		ATP1 (ISTp-AL– IPTp-SP)		0.029 (−0.053, 0.110)	0.49

Analysis population, Intervention group	No. ever had clinical malaria	No. children	Risk	Risk ratio^a^ (95 % CI)	p value*
ATP1, IPTp-SP	54	442	0.12	(Reference)	–
ATP1, ISTp-AL	68	440	0.15	1.27 (0.91, 1.76)	0.16
				Risk difference^a^ (95 % CI)	p value*
		ATP1 (ISTp-AL– IPTp-SP)		0.032 (−0.013,0.078)	0.17

*IPTp-SP* intermittent preventive treatment with sulfadoxine/pyrimethamine, *ISTp-AL* screening with a rapid diagnostic test (RDT) and treatment with artemether–lumefantrine, *ATP1* primary analysis according to protocol

^*^ Two-sided p values

^a^Covariates adjusted: for gender, socio-economic status, rural/urban residence location, irrigated area residence location, season, ITN use, age at visit, mother’s parasitaemia status on day of enrolment into the initial cohort, predelivery haemoglobin

**Fig. 3:**
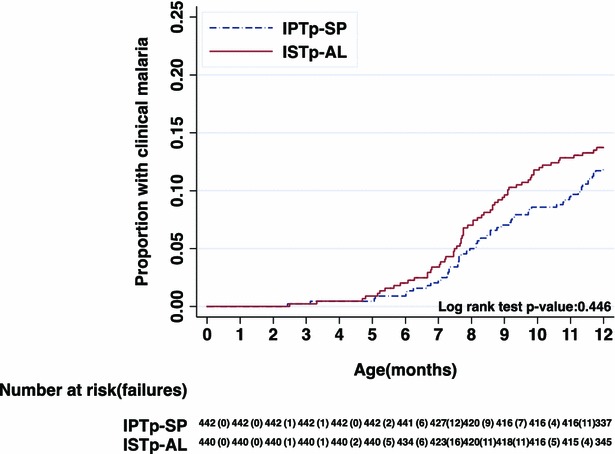
Cumulative incidence of clinical malaria episode by age—ATP1 population

Sixty-eight of the 493 children in the ISTp-AL group (15.5 %) and 54 of the 495 in the IPTp-SP group (12.2 %) experienced at least one passively detected episode of clinical malaria (adjusted risk ratio = 1.27 [95 % CI 0.91, 1.76]) (Table 1). Nelson-Aalen plots of cumulative incidence of clinical malaria showed similar incidence patterns in the two groups (Figs. 2, 3).

The incidence of all fevers among study children per year was 1.11 [95 % CI 1.03, 1.20] overall, and the rate ratio between the two study groups was 0.99 [95 % CI 0.83, 1.17] (Table 3). For fevers without malaria parasitaemia, the incidence rate per year was 0.83 [95 % CI 0.76, 0.90] and the rate ratio was 0.97 [95 % CI 0.83, 1.15]. The overall incidence of anaemia per year was 0.20, similar in both groups: 0.189 and 0.204 in the ISTp-AL and IPTp-SP groups, respectively; rate ratio 0.95 [95 % CI 0.83, 1.10].

**Table 3:** Incidence rates for fevers, non-malaria fevers and anaemia (all episodes during passive surveillance)

Analysis population, Intervention group	Fever episodes	Person-years at risk	Incidence rate per year	Rate ratio^a^ (95 % CI)	p value*
ATP1, IPTp-SP	356	312.7	1.14	(Reference)	–
ATP1, ISTp-AL	336	308.5	1.09	0.99 (0.83, 1.17)	0.88

Analysis population, Intervention group	Non-malaria fever episodes	Person-years at risk	Incidence rate per year	Rate ratio^a^ (95 % CI)	p value*
ATP1, IPTp-SP	262	312.7	0.84	(Reference)	–
ATP1, ISTp-AL	251	308.5	0.81	0.97 (0.83, 1.15)	0.74

Analysis population, Intervention group	Anaemia episodes	Person-years at risk	Incidence rate per year	Rate ratio^a^ (95 % CI)	p value*
ATP1, IPTp-SP	59	312.7	0.19	(Reference)	–
ATP1, ISTp-AL	63	308.5	0.20	0.95 (0.83, 1.10)	0.52

*IPTp-SP* intermittent preventive treatment with sulfadoxine/pyrimethamine, *ISTp-AL* screening with a rapid diagnostic test (RDT) and treatment with artemether–lumefantrine, *ATP1* primary analysis according to protocol

* Two-sided p values

^a^Covariates adjusted: for gender, socio-economic status, rural/urban residence location, irrigated area residence location, season, ITN use, age at visit, mother’s parasitaemia status on day of enrolment into the initial cohort, predelivery haemoglobin

In the ATP1 population, the overall prevalence of malaria parasitaemia at 6 months of age was 7.4 and 6.6 % in the ISTp-AL and IPTp-SP groups, respectively, prevalence ratio 1.16 (95 % CI 0.69, 1.95). At 12 months of age the overall prevalence of parasitaemia was 10.8 and 9.5 % in the ISTp-AL and IPTp-SP groups, respectively, prevalence ratio 1.18 (95 % CI 0.78, 1.78). The prevalence of anaemia was 68.0 % in the ISTp-AL group and 73.1 % in the IPTp-SP group at 6 months of age, prevalence ratio 0.94 (95 % CI 0.83, 1.07) and 84.7 and 83.1 % in the ISTp-AL and IPTp-SP groups, respectively, at 12 months of age, prevalence ratio 1.02 (95 % CI 0.95, 1.09). Similar results were found for the ATP2 population (Table 4).

**Table 4:** Prevalence of *Plasmodium falciparum* parasitaemia and anaemia at preplanned surveys at 6 and 12 months of age

Analysis population, Intervention group	No. ever had *P. falciparum* infection	No. children	Risk	Risk ratio^a^ (95 % CI)	p value*
Risk of *P*. *falciparum* infection at 6 months of age					
ATP1, IPTp-SP	25	379	0.066	(Reference)	–
ATP1, ISTp-AL	28	376	0.074	1.16 (0.69, 1.95)	0.56

Analysis population, Intervention group	No. ever had *P. falciparum* infection	No. children	Risk	Risk ratio^a^ (95 % CI)	p value*
Risk of *P*. *falciparum* infection at 12 months of age					
ATP1, IPTp-SP	38	402	0.095	(Reference)	–
ATP1, ISTp-AL	44	406	0.108	1.18 (0.78, 1.78)	0.44

Analysis population, Intervention group	No. ever had anaemia	No. children	Risk	Risk ratio^a^ (95 % CI)	p value*
Risk of anaemia (<11.0 g/dL) at 6 months of age					
ATP1, IPTp-SP	147	201	0.731	(Reference)	–
ATP1, ISTp-AL	138	203	0.680	0.94 (0.83, 1.07)	0.34

Analysis population, Intervention group	No. ever had anaemia	No. children	Risk	Risk ratio^a^ (95 % CI)	p value*
Risk of anaemia (<11.0 g/dL) at 12 months of age					
ATP1, IPTp-SP	265	319	0.830	(Reference)	–
ATP1, ISTp-AL	282	333	0.847	1.02 (0.95, 1.09)	0.59

*IPTp-SP* intermittent preventive treatment with sulfadoxine/pyrimethamine, *ISTp-AL* screening with a rapid diagnostic test (RDT) and treatment with artemether–lumefantrine, *ATP1* primary analysis according to protocol

* Two-sided p values

^a^Covariates adjusted: for gender, socio-economic status, rural/urban residence location, irrigated area residence location, season, ITN use, age at visit, mother’s parasitaemia status on day of enrolment into the initial cohort, predelivery haemoglobin

Among the 686 children (339 in the ISTp-AL group, 347 in the IPTp-SP group,) whose mothers’ PM status was known, the incidence of malaria and other study endpoints was similar among infants whose mothers had PM and infants whose mothers did not. The incidence of clinical malaria episodes in the PM-negative and PM-positive groups, respectively, were 0.22 per year and 0.20 per year, rate ratio 0.86 [95 % CI 0.54, 1.37]. The respective incidence rates in the PM-negative and PM-positive groups for non-malarial fevers were 0.90 per year and 0.73 per year with a rate ratio of 0.85 [95 % CI 0.69, 1.05]. For anaemia, respective incidence rates in the PM-negative and PM-positive groups were 0.16 per year and 0.20 per year, with an overall rate ratio of 0.99 [95 % CI 0.82, 1.20] (Table 5). Similar results for the ATP2 analyses are shown on the Additional files 3, 4, 5, 6.

**Table 5:** Incidence rates of clinical malaria, fever overall and non-malaria fevers in children born to women with or without placental malaria (all episodes during passive surveillance)

Analysis population, Intervention group	Clinical malaria episodes	Person-years at risk	Incidence rate per year	Rate ratio^a^ (95 % CI)	p value*
ATP1, PM−	68	303.0	0.22	(Reference)	–
ATP1, PM+	26	133.0	0.20	0.86 (0.54, 1.37)	0.52

Analysis population, Intervention group	Fever episodes	Person-years at risk	Incidence rate per year	Rate ratio^a^ (95 % CI)	p value*
ATP1, PM−	361	303.0	1.19	(Reference)	–
ATP1, PM+	131	133.0	0.98	0.90(0.72, 1.12)	0.33

Analysis population, Intervention group	Non-malaria fever episodes	Person-years at risk	Incidence rate per year	Rate ratio^a^ (95 % CI)	p value*
ATP1, PM−	272	303.0	0.90	(Reference)	–
ATP1, PM+	97	133.0	0.73	0.85 (0.69, 1.05)	0.13

Analysis population, Intervention group	Anaemia episodes	Person-years at risk	Incidence rate per year	Rate ratio^a^ (95 % CI)	p value*
ATP1, PM−	49	303.0	0.16	(Reference)	–
ATP1, PM+	26	133.0	0.20	0.99 (0.82, 1.20)	0.94

*IPTp-SP* intermittent preventive treatment with sulfadoxine/pyrimethamine, *ISTp-AL* screening with a rapid diagnostic test (RDT) and treatment with artemether–lumefantrine, *ATP1* primary analysis according to protocol

* Two-sided p values

^a^Covariates adjusted: for gender, socio-economic status, rural/urban residence location, irrigated area residence location, season, ITN use, age at visit, mother’s parasitaemia status on day of enrolment into the initial cohort, predelivery haemoglobin

**Fig. 4:**
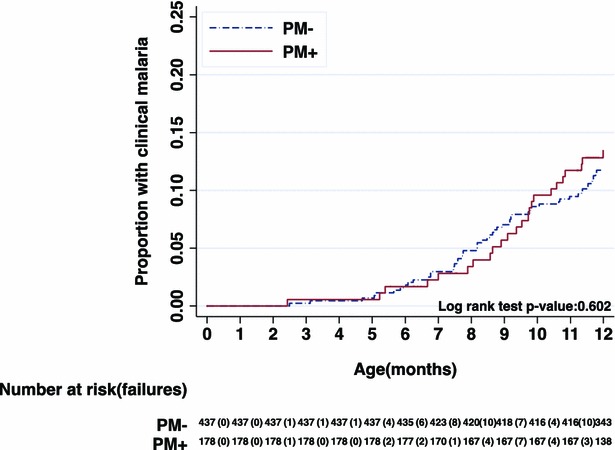
Cumulative incidence of clinical malaria episode among off springs of placental malaria negative (PM−) and placental malaria positive (PM+) mothers—ATP1 population

**Fig. 5:**
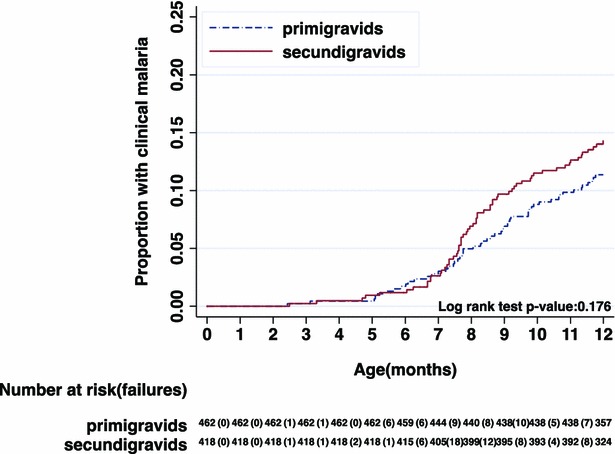
Cumulative incidence of clinical malaria episode among off springs of primigravids and secundigravids—ATP1 population

Nelson-Aalen plots of cumulative incidence of clinical malaria among infants of mother’s PM positive or negative and gravidity status also showed similarity in the incidence malaria (Figs. 4, 5), respectively.

## Discussion

The findings of this study suggest that children born to women who were managed with ISTp-AL during ANC visits did not experience an elevated risk of clinical malaria compared to those born to mothers who were given IPTp-SP. The incidence of all febrile illnesses, non-malaria fever and anaemia was also similar in the two groups of children. This finding may be attributable to the randomization of a large number of women to IPTp-SP or ISTp-AL groups successfully balancing factors related to exposure of the mother, and consequently the exposure of the infant, and because the two interventions performed similarly well in preventing PM.

The risk of malaria in infancy was not increased in the infants of women who had PM, agreeing with some previous studies that investigated the risk among primigravidae [2] or in all gravidities [6], but not with other studies which found an elevated risk [3, 4, 25]. Why this finding varies from study to study is uncertain. It is possible that apparent elevated incidence in infants whose mothers had PM is a consequence of confounding by exposure, or at least partly due to this, and that this effect is stronger in some situations than in others. In this study, adjusting for mothers’ parasitaemia at enrolment, gender, place of residence, socio-economic status, and ITN use did not identify any strong sources of confounding.

This study had a number of limitations. As there was a delay in obtaining the resources needed to conduct the study, not all infants were recruited immediately after birth and the time period during which the one-year period of follow-up took place varied from nought–12 to 5–17 months of age. However, the age pattern of follow-up was very similar in the two groups. In addition, 18.7 % of women in the initial cohort and their infants were not enrolled for follow-up in this study, primarily because some mothers who had delivered in the main study could not be located when the infant follow-up began. Randomization of infants was dictated by the study group of their mothers and not by randomization of the infants themselves, but the baseline characteristics of the infants in each study group were well balanced. The sample size of the infant population was driven by the number of women enrolled into the pregnancy trial and could not be adjusted for the purpose of this follow-up study. The study was planned to have sufficient power to exclude a relative increase in the incidence of malaria of 20 % between the two groups, assuming the malaria incidence rates previously found in the study area. However, the much lower than expected incidence of malaria meant that it was not possible to rule out differences of this magnitude between the ISTp-AL and IPTp-SP groups.

## Conclusion

There are currently no grounds for changing the well-accepted WHO policy of administration of SP at each routine ANC attendance in the study area, or in other areas where *P. falciparum* is still sensitive to SP. However, if ISTp-AL is to be deployed in the future in any specific epidemiological situation, the results of this study suggest that infants will not be put at any major increased risk.

## Additional files

10.1186/s12936-016-1094-z Characteristics of study mothers and children who were enrolled or not enrolled. Analysis of the data showing the characteristics of mothers and children who were enrolled or not enrolled into the study.
10.1186/s12936-016-1094-z The number of all infants followed up by study arm, duration (months) and age at enrolment (months). Analysis of the data showing the number of children enrolled at various ages in months, duration of followed time in months by the study arm. 
10.1186/s12936-016-1094-z Incidence of episodes of clinical malaria in study children (all episodes during passive surveillance)-ATP2 population. Statistical analysis of the data showing the incidence of all clinical malaria episodes captured passively for the ATP2 population is presented on this table. Rate difference and the risk of clinical malaria comparing the children in the two study groups have also been presented. 
10.1186/s12936-016-1094-z Incidence rates for fevers, non-malaria fevers and anaemia- ATP2 population. Statistical analysis of the data showing incidence rates for fevers, non-malaria fevers and anaemia episodes captured passively for the ATP2 population is presented on this table. 
10.1186/s12936-016-1094-z Prevalence of P. falciparum parasitaemia and anaemia at pre-planned surveys at 6 and 12 months of age - ATP2 population. Statistical analysis of the data showing risk of Plasmodium falciparum parasitaemia and anaemia (Hb<11g/dL) at pre-planned surveys at 6 and 12 months of age are presented for the ATP2 population. 
10.1186/s12936-016-1094-z Incidence rates of clinical malaria, fever overall and non-malaria fevers in children born to women with or without placental malaria (PM) - ATP2 population. Statistical analysis of the data showing incidence rates of clinical malaria, fever overall and non-malaria fevers in children born to women with or without placental malaria (PM) for all episodes during passive surveillance for the ATP2 population.
