# Supplementary material for: The risk of malaria in Ghanaian infants born to women managed in pregnancy with intermittent screening and treatment for malaria or intermittent preventive treatment with sulfadoxine/pyrimethamine
Source: Malar J. 2016 Jan 28;15:46. doi: 10.1186/s12936-016-1094-z (PMC4730594; doi:10.1186/s12936-016-1094-z)
Supplement: Supplementary file 1 — 10.1186/s12936-016-1094-z Characteristics of study mothers and children who were enrolled or not enrolled. Analysis of the data showing the characteristics of mothers and children who were enrolled or not enrolled into the study. [file 12936_2016_1094_MOESM1_ESM.docx]

**Table S1.** Characteristics of study mothers and children who were enrolled or not enrolled.

| **Characteristics** | **Enrolled**  n(988) | **Not Enrolled**  n(238) |
| --- | --- | --- |
| **Maternal** |  |  |
|  |  |  |
| **Gravidity**, n (%) |  |  |
| Primi | 528(53.5) | 157(66.5) |
| Secondi | 459(46.5) | 79(33.5) |
| **Number of IPTp-SP or ISTp-AL,** n (%) |  |  |
| 1 | 72(7.3) | 27(11.4) |
| 2 | 274(27.7) | 71(30.0) |
| 3 | 643(65.0) | 137(57.8) |
| **Placental Malaria (PM),** n^*^ (%) |  |  |
| PM+ | 203(70.4) | 47(68.9) |
| PM- | 483(29.6) | 104(31.1) |
| **Marital status,** n (%) |  |  |
| Married | 865(87.6) | 198(83.5) |
| Single | 120(12.1) | 35(14.8) |
| Other | 3(0.3) | 4(1.7) |
| **Age at delivery** [mean (SD)],years | 22.3(3.7) | 21.7(4.9) |
|  |  |  |
| **SES,** n^*^ (%) |  |  |
| Least poor | 112(11.4) | 42(17.8) |
| Less poor | 122(12.4) | 36(15.3) |
| Poor | 210(21.3) | 47(19.9) |
| More poor | 352(35.7) | 71(30.1) |
| Most poor | 190(19.3) | 40(17.0) |
| **Child** |  |  |
|  |  |  |
| **Gender,** n (%) |  |  |
| Male | 480(48.5) | 137(57.8) |
| Female | 508(51.5) | 100(42.2) |
| **Birth season,** n (%) |  |  |
| Wet(June to October) | 447(45.2) | 111(46.8) |
| Dry(November to May) | 542(54.8) | 126(53.2) |
| **Residence location,** n (%) |  |  |
| Urban | 108(10.9) | 36(15.2) |
| Rural | 880(89.1) | 201(84.8) |
| **Live in Irrigation area,** n (%) |  |  |
| Yes | 117(11.8) | 16(6.8) |
| No | 872(88.2) | 221(93.3) |
| **Birth weight** [mean(SD)],kg | 2.78(0.4) | 2.6(0.6) |

**PM=**Placental malaria**; IPTp-SP=** Intermittent preventive treatment with sulfadoxine/pyrimethamine **;**

**ISTp-AL=**Screening with a rapid diagnostic test (RDT) and treatment with artemether-lumefantrine

**SES=**Socio-economic status**; ITN=**Insecticide treated bednet**; SD=**Standard deviation
