# Supplementary material for: The risk of malaria in Ghanaian infants born to women managed in pregnancy with intermittent screening and treatment for malaria or intermittent preventive treatment with sulfadoxine/pyrimethamine
Source: Malar J. 2016 Jan 28;15:46. doi: 10.1186/s12936-016-1094-z (PMC4730594; doi:10.1186/s12936-016-1094-z)
Supplement: Supplementary file 2 — 10.1186/s12936-016-1094-z The number of all infants followed up by study arm, duration (months) and age at enrolment (months). Analysis of the data showing the number of children enrolled at various ages in months, duration of followed time in months by the study arm. [file 12936_2016_1094_MOESM2_ESM.docx]

**Table S2.**The number of all infants followed up by study arm, duration (months) and age at enrolment (months) .

| **Follow up  time (months)** | **IPTp-SP**  **n(495)** | | **ISTp-AL**  **n(493)** | |
| --- | --- | --- | --- | --- |
|  |  |  |  |  |
|  | **Age at enrolment** | | **Age at enrolment** | |
|  | <6months | >6months | <6months | >6months |
| 0-11 | 19 | 22 | 15 | 18 |
| 0-12 | 248 | 38 | 240 | 37 |
| 19-21 | 95 | 73 | 122 | 61 |
| Total | 362 | 133 | 377 | 116 |

**IPTp-SP=** Intermittent preventive treatment with sulfadoxine/pyrimethamine **;**

**ISTp-AL=**Screening with a rapid diagnostic test (RDT) and treatment with artemether-lumefantrine
