# Supplementary material for: The risk of malaria in Ghanaian infants born to women managed in pregnancy with intermittent screening and treatment for malaria or intermittent preventive treatment with sulfadoxine/pyrimethamine
Source: Malar J. 2016 Jan 28;15:46. doi: 10.1186/s12936-016-1094-z (PMC4730594; doi:10.1186/s12936-016-1094-z)
Supplement: Supplementary file 3 — 10.1186/s12936-016-1094-z Incidence of episodes of clinical malaria in study children (all episodes during passive surveillance)-ATP2 population. Statistical analysis of the data showing the incidence of all clinical malaria episodes captured passively for the ATP2 population is presented on this table. Rate difference and the risk of clinical malaria comparing the children in the two study groups have also been presented. [file 12936_2016_1094_MOESM3_ESM.docx]

**Table S3.** Incidence of episodes of clinical malaria in study children (all episodes during passive surveillance)-ATP2 population.

| **Analysis population, Intervention group** | **Clinical malaria episodes** | **Person-years at risk** | **Incidence rates**  **per year** | | **Rate ratio^a^**  **(95% CI)** | **p-value^*^** |
| --- | --- | --- | --- | --- | --- | --- |
| ATP2, IPTp-SP | 72 | 343.0 | 0.21 | | (reference) | - |
| ATP2, ISTp-AL | 77 | 331.5 | 0.23 | | 0.84 (0.61, 1.17) | 0.31 |
|  |  |  | | **Rate difference^a^ (95% CI)** | | **p-value** |
|  |  | ATP2 (ISTp-AL – IPTp-SP) | | 0.031 (-0.051, 0.112) | | 0.46 |
|  |  |  | |  | |  |
| **Analysis population,**  **Intervention group** | **No. ever had**  **clinical malaria** | **No. of children** | **Risk** | **Risk ratio^a^**  **(95% CI)** | | **p-value^*^** |
| ATP2, IPTp-SP | 59 | 495 | 0.12 | (reference) | | - |
| ATP2, ISTp-AL | 72 | 493 | 0.15 | 1.23 (0.89, 1.69) | | 0.21 |
|  |  |  | | | **Risk difference^a^ (95% CI)** | **p-value^*^** |
|  |  | ATP2 (ISTp-AL – IPTp-SP) | | | 0.027 (-0.015, 0.070) | 0.21 |

**IPTp-SP=** Intermittent preventive treatment with sulfadoxine/pyrimethamine **;**

**ISTp-AL=**Screening with a rapid diagnostic test (RDT) and treatment with artemether-lumefantrine

**ATP2** =Secondary analysis without strict adherence to protocol

*^a^covariates adjusted: for gender, socio-economic status , rural/urban residence location, irrigated area residence location, season, ITN use, age at visit, mother’s parasitaemia status on day of enrolment into the initial cohort, pre delivery haemoglobin*

*^*^ two sided p-values*
