# Supplementary material for: The risk of malaria in Ghanaian infants born to women managed in pregnancy with intermittent screening and treatment for malaria or intermittent preventive treatment with sulfadoxine/pyrimethamine
Source: Malar J. 2016 Jan 28;15:46. doi: 10.1186/s12936-016-1094-z (PMC4730594; doi:10.1186/s12936-016-1094-z)
Supplement: Supplementary file 4 — 10.1186/s12936-016-1094-z Incidence rates for fevers, non-malaria fevers and anaemia- ATP2 population. Statistical analysis of the data showing incidence rates for fevers, non-malaria fevers and anaemia episodes captured passively for the ATP2 population is presented on this table. [file 12936_2016_1094_MOESM4_ESM.docx]

**Table S4.** Incidence rates for fevers, non-malaria fevers and anaemia (all episodes during passive surveillance)-ATP2 population.

| **Analysis population, Intervention group** | **Fever episodes** | **Person-years at risk** | **Incidence rate**  **per year** | **Rate ratio^a^ (95% CI)** | **p-value^*^** |
| --- | --- | --- | --- | --- | --- |
| ATP2, IPTp-SP | 380 | 343.0 | 1.11 | (reference) | - |
| ATP2, ISTp-AL | 366 | 331.5 | 1.10 | 0.99 (0.84, 1.17) | 0.91 |
|  |  |  |  |  |  |
| **Analysis population, Intervention group** | **Non-malaria fever episodes** | **Person-years at risk** | **Incidence rate**  **per year** | **Rate ratio^a^ (95% CI)** | **p-value^*^** |
| ATP2, IPTp-SP | 282 | 343.0 | 0.82 | (reference) | - |
| ATP2, ISTp-AL | 273 | 331.5 | 0.82 | 0.96 (0.82, 1.12) | 0.64 |
|  |  |  |  |  |  |
| **Analysis population, Intervention group** | **Anaemia episodes** | **Person-years at risk** | **Incidence rate**  **per year** | **Rate ratio^a^ (95% CI)** | **p-value^*^** |
| ATP2, IPTp-SP | 67 | 343.0 | 0.20 | (reference) | - |
| ATP2, ISTp-AL | 68 | 331.5 | 0.21 | 0.93 (0.81, 1.07) | 0.33 |

**IPTp-SP=** Intermittent preventive treatment with sulfadoxine/pyrimethamine **;**

**ISTp-AL=**Screening with a rapid diagnostic test (RDT) and treatment with artemether-lumefantrine

**ATP2** =Secondary analysis without strict adherence to protocol

*^a^covariates adjusted: for gender, socio-economic status , rural/urban residence location, irrigated area residence location, season, ITN use, age at visit, mother’s parasitaemia status on day of enrolment into the initial cohort, pre delivery haemoglobin*

*^*^ two sided p-values*
