# Supplementary material for: The risk of malaria in Ghanaian infants born to women managed in pregnancy with intermittent screening and treatment for malaria or intermittent preventive treatment with sulfadoxine/pyrimethamine
Source: Malar J. 2016 Jan 28;15:46. doi: 10.1186/s12936-016-1094-z (PMC4730594; doi:10.1186/s12936-016-1094-z)
Supplement: Supplementary file 5 — 10.1186/s12936-016-1094-z Prevalence of P. falciparum parasitaemia and anaemia at pre-planned surveys at 6 and 12 months of age - ATP2 population. Statistical analysis of the data showing risk of Plasmodium falciparum parasitaemia and anaemia (Hb<11g/dL) at pre-planned surveys at 6 and 12 months of age are presented for the ATP2 population. [file 12936_2016_1094_MOESM5_ESM.docx]

**Table S5**. Prevalence of *Plasmodium falciparum* parasitaemia and anaemia at pre-planned surveys at 6 and 12 months of age -ATP2 population.

| **Risk of *P*.*falciparum* infection at 6 months of age** | | | | | |
| --- | --- | --- | --- | --- | --- |
| **Analysis population, Intervention group** | **No. ever had *P. falciparum* infection** | **No of children** | **Risk** | **Risk ratio^a^**  **(95% CI)** | **p-value^*^** |
| ATP2, IPTp-SP | 26 | 410 | 0.063 | (reference) | - |
| ATP2, ISTp-AL | 31 | 402 | 0.077 | 1.20 (0.73, 1.20) | 0.46 |
| **Risk of *P*.*falciparum* infection at 12 months of age** | | | | | |
| **Analysis population, Intervention group** | **No. ever had *P. falciparum* infection** | **No of children** | **Risk** | **Risk ratio^a^**  **(95% CI)** | **p-value^*^** |
| ATP2, IPTp-SP | 40 | 438 | 0.091 | (reference) | - |
| ATP2, ISTp-AL | 49 | 433 | 0.113 | 1.23 (0.83, 1.84) | 0.30 |
| **Risk of anaemia (<11.0g/dL) at 6 months of age** | | | | | |
| **Analysis population, Intervention group** | **No. ever had anaemia** | **No of children** | **Risk** | **Risk ratio^a^**  **(95% CI)** | **p-value^*^** |
| ATP2, IPTp-SP | 159 | 223 | 0.713 | (reference) | - |
| ATP2, ISTp-AL | 148 | 219 | 0.676 | 0.94 (0.84, 1.07) | 0.40 |
| **Risk of anaemia (<11.0g/dL) at 12 months of age** | | | | | |
| **Analysis population, Intervention group** | **No. ever had anaemia** | **No of children** | **Risk** | **Risk ratio^a^**  **(95% CI)** | **p-value^*^** |
| ATP2, IPTp-SP | 287 | 350 | 0.820 | (reference) | - |
| ATP2, ISTp-AL | 298 | 353 | 0.844 | 1.02 (0.96, 1.09) | 0.47 |

**IPTp-SP=** Intermittent preventive treatment with sulfadoxine/pyrimethamine **;**

**ISTp-AL=**Screening with a rapid diagnostic test (RDT) and treatment with artemether-lumefantrine

**ATP2** =Secondary analysis without strict adherence to protocol

*^a^covariates adjusted : gender, socio-economic status , rural/urban residence location, irrigated area residence location, season, ITN use, age at visit, mother’s parasitaemia status on day of enrolment into the initial cohort , pre delivery haemoglobin*

*^*^ two sided p-value*
