# Supplementary material for: The risk of malaria in Ghanaian infants born to women managed in pregnancy with intermittent screening and treatment for malaria or intermittent preventive treatment with sulfadoxine/pyrimethamine
Source: Malar J. 2016 Jan 28;15:46. doi: 10.1186/s12936-016-1094-z (PMC4730594; doi:10.1186/s12936-016-1094-z)
Supplement: Supplementary file 6 — 10.1186/s12936-016-1094-z Incidence rates of clinical malaria, fever overall and non-malaria fevers in children born to women with or without placental malaria (PM) - ATP2 population. Statistical analysis of the data showing incidence rates of clinical malaria, fever overall and non-malaria fevers in children born to women with or without placental malaria (PM) for all episodes during passive surveillance for the ATP2 population. [file 12936_2016_1094_MOESM6_ESM.docx]

**Table S6.** Incidence rates of clinical malaria, fever overall and non-malaria fevers in children born to women with or without placental malaria (PM) (all episodes during passive surveillance)- ATP2 population.

| **Analysis population, Intervention group** | **Clinical malaria episodes** | **Person-years at risk** | **Incidence rate**  **per year** | **Rate ratio^a^**  **(95% CI)** | **p-value^*^** |
| --- | --- | --- | --- | --- | --- |
| ATP2, PM- | 74 | 324.1 | 0.22 | (reference) | - |
| ATP2, PM+ | 28 | 147.8 | 0.19 | 0.90 (0.57, 1.41) | 0.63 |
|  |  |  |  |  |  |
| **Analysis population, Intervention group** | **Fever episodes** | **Person-years at risk** | **Incidence rate**  **per year** | **Rate ratio^a^**  **(95% CI)** | **p-value^*^** |
| ATP2, PM- | 381 | 324.1 | 1.18 | (reference) | - |
| ATP2, PM+ | 149 | 147.8 | 1.00 | 0.90(0.72, 1.11) | 0.31 |
|  |  |  |  |  |  |
| **Analysis population, Intervention group** | **Non-malaria**  **fever episodes** | **Person-years at risk** | **Incidence rate**  **per year** | **Rate ratio^a^**  **(95% CI)** | **p-value^*^** |
| ATP2, PM- | 286 | 324.1 | 0.88 | (reference) | - |
| ATP2, PM+ | 113 | 147.8 | 0.76 | 0.88 (0.72, 1.09) | 0.24 |
|  |  |  |  |  |  |
| **Analysis population, Intervention group** | **Anaemia episodes** | **Person-years at risk** | **Incidence rate**  **per year** | **Rate ratio^a^**  **(95% CI)** | **p-value^*^** |
| ATP2, PM- | 53 | 324.1 | 0.16 | (reference) | - |
| ATP2, PM+ | 31 | 147.8 | 0.21 | 0.97 (0.81, 1.17) | 0.76 |

**IPTp-SP=** Intermittent preventive treatment with sulfadoxine/pyrimethamine **;**

**ISTp-AL=**Screening with a rapid diagnostic test (RDT) and treatment with artemether-lumefantrine

**ATP2** =Secondary analysis without strict adherence to protocol

**PM**+=Placental malaria positive

**PM**-=Placental malaria negative

*^a^covariates adjusted: gender, socio-economic status , rural/urban residence location, irrigated area residence location, season, ITN use, age at visit, mother’s parasitaemia status on day of enrolment into the initial cohort, pre delivery haemoglobin*

*^*^ two sided p-values*
